# The Role of Cu Length on the Magnetic Behaviour of Fe/Cu Multi-Segmented Nanowires

**DOI:** 10.3390/nano8070490

**Published:** 2018-07-04

**Authors:** Suellen Moraes, David Navas, Fanny Béron, Mariana P. Proenca, Kleber R. Pirota, Célia T. Sousa, João P. Araújo

**Affiliations:** 1 Instituto de Física dos Materiais da Universidade do Porto—Instituto de Nanotecnologia and Department Física e Astronomia da Faculdade de Ciências da Universidade do Porto, Rua do Campo Alegre 687, 4169-007 Porto, Portugal; suellen.fisica@gmail.com (S.M.); davidnavasotero@gmail.com (D.N.); marianapproenca@gmail.com (M.P.P.); jearaujo@fc.up.pt (J.P.A.); 2 Instituto de Física Gleb Wataghin (IFGW), Universidade Estadual de Campinas (UNICAMP), Campinas, SP 13083-859, Brazil; fberon@ifi.unicamp.br (F.B.); krpirota@ifi.unicamp.br (K.R.P.); 3 Instituto de Sistemas Optoelectrónicos y Microtecnología, Universidad Politécnica de Madrid, Avda., Complutense 30, E-28040 Madrid, Spain

**Keywords:** multi-segmented nanowires, magnetic interactions, FORC, micromagnetic simulations

## Abstract

A set of multi-segmented Fe/Cu nanowires were synthesized by a two-step anodization process of aluminum substrates and a pulsed electrodeposition technique using a single bath. While both Fe segment length and diameter were kept constant to (30 ± 7) and (45 ± 5) nm, respectively, Cu length was varied between (15 ± 5) and (120 ± 10) nm. The influence of the non-magnetic layer thickness variation on the nanowire magnetic properties was investigated through first-order reversal curve (FORC) measurements and micromagnetic simulations. Our analysis confirmed that, in the multi-segmented Fe/Cu nanowires with shorter Cu segments, the dipolar coupling between Fe segments controls the nanowire magnetic behavior, and its performance is like that of a homogenous Fe nanowire array of similar dimensions. On the other hand, multi-segmented Fe/Cu nanowires with larger Cu segments act like a collection of non-interacting magnetic entities (along the nanowire axis), and their global behavior is mainly controlled by the neighbor-to-neighbor nanodisc dipolar interactions.

## 1. Introduction

Ferromagnetic nanostructures with complex and controlled magnetic behavior have been extensively studied during the last years. The need for low-energy technologies and the recent advances in chemical and self-assembly synthesis techniques have boosted the growth of three-dimensional nano-objects [1]. This capacity to control size and shape, not only in the plane but also along the vertical direction, leads to the appearance of new effects in spin configurations [2,3]. This potential is of great interest for a wide range of applications, such as spintronics and novel magnetic sensors [4,5], chemical and biological sensors [6], drug delivery systems [7] and medical treatment by hyperthermia [8].

Within several methods of nanowire (NW) fabrication, template-assisted electrodeposition in anodic aluminum oxide (AAO) has been widely used [9,10,11] because of its ability to control the NW size and shape, coupled with simplicity and low cost of the involved processes. Moreover, the NWs inside AAO are organized in a closed-package array, which allows the better understanding of the inter-wire and intra-wire magnetic interactions [12]. 

An important class of NWs for applications in magnetic devices is the multi-segmented [13,14] one. The combination of multilayers of different materials, both magnetic and non-magnetic, has been spreading much interest due to the magnetic capability to control the interactions and the effect of magnetic anisotropy obtained by only varying the metal type and segment lengths [15,16,17]. In this sense, we can highlight some studies on multi-segmented NWs that employed Ni/Au [18,19], Ni/Cu [13,20,21,22,23,24,25,26], CoNi/Cu [27], NiFe/Cu [28], FeGa/Cu [16,17], Co/Cu [29,30,31,32], and Co/Au [33]. Because of particular advantages, such as their high saturation magnetization [34] and the large possibility of modulation of their magnetic properties [35], systems with FeCo alloys (i.e., CoFeB/Cu [36], CoFe/Au [35], CoFe/Cu [37,38,39,40,41]) have been well studied. Núñez et al. [37] investigated the magnetic behavior of CoFe/Cu multi-segmented NWs by varying the Cu spacer thickness. Complementarily, Palmero et al. [39] presented a study of first-order reversal curve (FORC) diagrams for CoFe/Cu multi-segmented NWs. However, for some applications, namely biomedical, Co is considered toxic, and, thus, a magnetic segment with only Fe in its composition could be more interesting. Still, only a few studies are found in the literature about Fe/Cu multi-segmented NWs: Ramazani et al. [42] presented a comparative study between the multi-segmented NWs of Co/Cu, CoFe/Cu, and Fe/Cu, and Almasi-Kashi et al. [43] studied the effect of the magnetic layer thickness on the magnetic properties of Fe/Cu multi-segmented NWs. Moreover, most of these works are aimed for applications in electronic devices, leaving open the biotechnology field. In this field, the main challenge is to achieve a better control and to understand the magnetic behavior of 3D magnetic systems composed only of purely biocompatible materials that enable future applications. 

In this context, to better understand the magnetic interactions of these biocompatible NWs confined into the pores of AAO templates, the FORC technique has proved to be very effective. This technique consists in the acquisition of several minor hysteresis curves beginning at different reversal fields. In this work, we present the synthesis and characterization of Fe/Cu multi-segmented NWs obtained from pulsed electrodeposition in AAO templates. The magnetic properties of the NW arrays with different Cu segments were extensively studied to understand the role of interlayer spacing.

## 2. Materials and Methods 

The Fe/Cu multi-segmented NWs were fabricated by electrochemical deposition from a single aqueous bath using AAO as a template. AAO membranes were prepared from high-purity (>99.999%) Al foils by a standard two-step anodization method [44]. The Al foils were cleaned by means of ultrasonication in acetone and ethanol and then electropolished in a 75% ethanol/25% perchloric acid solution at 20 V for 2 min to reduce the surface roughness and create nanopatterns for subsequent pore nucleation [45]. The first and second anodization lasted 24 h and 48 h, respectively, at a constant voltage of 40 V in a 0.3 M oxalic acid with a controlled temperature of 5 ± 1 °C. By this process, self-organized AAO templates were obtained with 1 cm in diameter, 120 µm in thickness, and typical pore diameter and interpore distances of *d* ≈ 35 ± 5 nm and *D*_int_ ≈ 105 ± 5 nm, respectively.

After the anodization process, the Al substrate was removed in a 0.2 M CuCl_2_, 4.1 M HCl aqueous solution, at room temperature. The released AAO was floated in 0.5 M H_3_PO_4_ at room temperature, to remove the nanopore bottom and also increase the pore diameter to (45 ± 5) nm. An Au film layer was sputtered to provide a conductive metallic contact at the pore bottom. The electrodeposition was performed in a three-electrode cell equipped with an Ag/AgCl reference electrode, a Pt mesh as a counter electrode, and the gold-coated AAO membrane acting as the working electrode. Before the NW electrodeposition, the Au layer was thickened by electrodepositing a commercial gold-plating solution (Orosene E + 4gr/lt, pH 3.5 − 4.5, from Italgalvano) at −1.0 V during 5 min.

The Fe/Cu NWs were grown at room temperature by the DC pulsed electrodeposition method. The used electrolyte contained 0.4 M H_3_BO_3_, 0.1 M FeSO_4_·7H_2_O, 0.005 M CuSO_4_·5H_2_O, and 0.003 M ascorbic acid. The multilayer deposition was carried out with a sequence of pulses at −1.1 V to grow the Fe layer, followed by a pulse at an optimized potential of −0.6 V to grow the Cu layer. These pulses were repeatedly applied until achieving 15 bilayers of Fe/Cu. The deposition rate was calibrated through the electrodeposition of long NWs of each metal, accordingly with each potential (≈2.5 nm/s at −1.1 V for Fe, and ≈ 0.3 nm/s at −0.6 V for Cu), using the composition of the pulsed electrodeposition electrolyte, thus accurately controlling the length (*L*) of each segment.

The wire morphology was analyzed by scanning electron microscopy (SEM) using a FEI Inspect F50 microscope (FEI Europe BV, Madrid, Spain) The crystallographic structure of the Fe NWs was investigated by X-ray powder diffraction (XRD) using a Rigaku SmartLab diffractometer (Rigaku Corporation, Tokio, Japan) with Cu-Kα radiation (1.540593 Å), 45 kV and 200 mA. The data were collected at room temperature and ranged between 35° ≤ 2θ ≤ 90° in a Bragg Brentano geometry, with 0.02° steps. The magnetic hysteresis loops [*M*(*H*)] were measured with the field (*H*) applied along the parallel (*H*^ǁ^) and perpendicular (*H*^┴^) directions with respect to the NWs’ longitudinal axis in a vibrating sample magnetometer (VSM), LakeShore Controller Model 7304 (Lake Shore Cryotronics Inc., Westerville, OH, USA), at room temperature. Moreover, the first-order reversal curves (FORCs) were acquired for a parallel applied magnetic field. After saturating the nanowire array at 10 kOe, 100 minor (reversal) curves were measured, covering the ± 2500 Oe region. Each curve was preceded by a saturation and consisted of a 25 Oe field spacing. The FORC distribution was calculated as the second-order mixed derivative of the magnetization, with respect to the reversal field (*H*_r_) and applied field (*H*) [46]. The results are presented in a Preisach plane, with the diagonal axes being *H*_c_ = 0.5 (*H* − *H*_r_) and *H*_u_ = −0.5 (*H* + *H*_r_). The distribution strength is given through the color scale, ranging from blue (null value) to red (maximum value).

## 3. Results

### 3.1. Morphological Characterization

**Figure 1:**
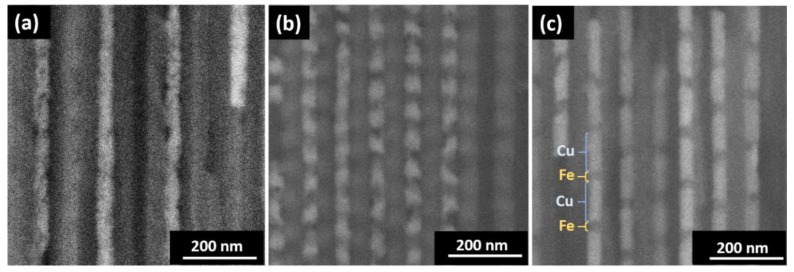
Cross-sectional Scanning Electron Microscopy (SEM) images of Fe/Cu nanowires (NWs) grown in Anodic Aluminum Oxide (AAO) membranes. The average diameter and length of the Fe segments were kept constant to (45 ± 5) nm and (30 ± 7) nm, respectively, while the Cu segment length (*L*_Cu_) was varied: (**a**) (15 ± 5) nm; (**b**) (60 ± 6) nm; (**c**) (120 ± 10) nm.

SEM images, shown in Figure 1, display selected cross-sectional views of the Fe/Cu NWs grown in the AAO membranes. The layered structure and layer thickness homogeneity are clearly observed, in which brighter segments are composed of Cu, while the darker ones correspond to Fe layers. The NWs presented an average diameter of (45 ± 5) nm, mimicking the pore size of the AAO template. In this work, the Cu segments’ length (*L*_Cu_) was varied, whereas the Fe electrodeposition parameters were maintained constant in all samples. As a result, the Fe segments length (*L*_Fe_) presented an average of (30 ± 7) nm, and *L*_Cu_ varied from (15 ± 5) nm (Figure 1a) to (120 ± 10) nm (Figure 1c).

### 3.2. Structural Characterization 

**Figure 2:**
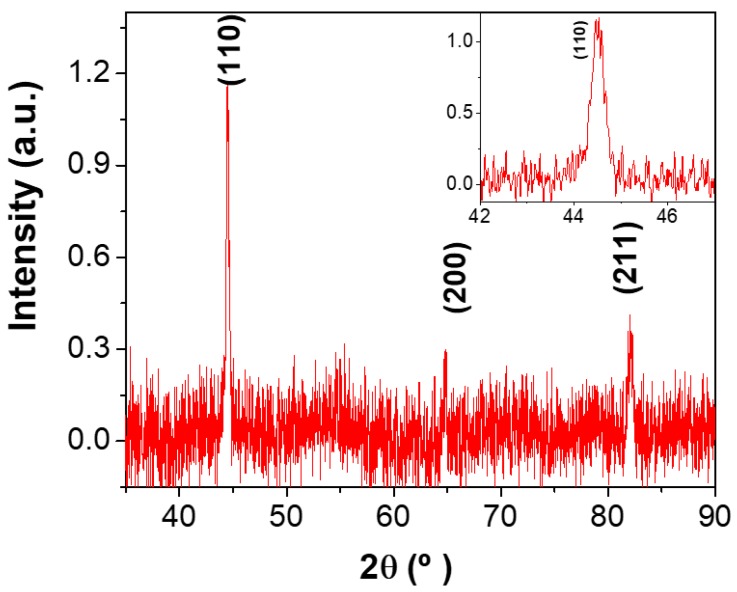
X-ray diffractometry (XRD) pattern of an electrodeposited Fe NW array. The inset shows a magnified view of the XRD pattern around the (110) crystallographic peak.

XRD analysis was made for Fe NWs with (3.2 ± 0.2) µm in length electrodeposited in AAO templates with a similar electrolyte to that used for Fe/Cu multi-segmented NWs. The Fe NWs presented a polycrystalline body-centered cubic (bcc) structure (Figure 2). This configuration exhibited one main peak and two secondary peaks with lower intensities at 44.52°, 65.04°, and 82.12°, which corresponded to the (110), (200), and (220) crystallographic planes, respectively. Therefore, the Fe NWs had grown in a polycrystalline bcc structure with (110) texture.

### 3.3. Magnetic Characterization

**Figure 3:**
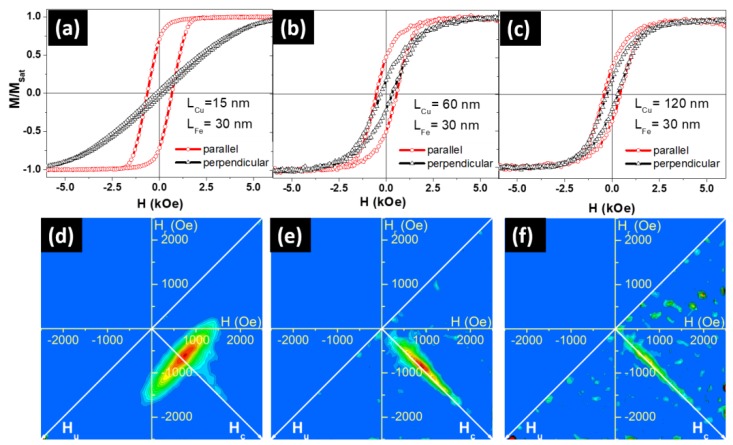
Hysteresis loops of multi-segmented Fe/Cu NWs when the external magnetic field was applied parallel (in red) and perpendicular (in black) to the NW long axis and for *L*_Cu_ (**a**) 15 nm, (**b**) 60 nm, and (**c**) 120 nm. Their related first-order reversal curve (FORC) diagrams when the external magnetic field was applied parallel to the NWs’ long axis and for *L*_Cu_ (**d**) 15 nm, (**e**) 60 nm, and (**f**) 120 nm.

Figure 3 shows the magnetic hysteresis loops (Figure 3a–c) and their related FORC diagrams (Figure 3d–f) of the electrodeposited Fe/Cu NW arrays. Room temperature hysteresis loops were measured by applying the external magnetic field parallel (in red) and perpendicular (in black) to the NW long axis. Both coercive fields (*H*_c_) and reduced remanence ($m_{r}$), given by the ratio M_r_/M_sat_ (where M_r_ is the magnetization remanence and M_sat_ is the saturation magnetization), are summarized in Table 1. A decrease (increase) of the parallel coercivity $H_{c}^{\left|\right|},$ (perpendicular coercivity $H_{c}^{⊥}$ was observed when the *L*_Cu_ was enlarged (Table 1). The same behavior was obtained for the parallel and perpendicular reduced remanence ($m_{r}$).

Therefore, and as a general idea extracted from the hysteresis loops, we could conclude that the easy magnetization axis evolved from parallel to the NW long axis for the Fe NWs with the thinnest Cu segments (*L*_Cu_ = 15 nm), to an almost isotropic behavior for the thickest case (*L*_Cu_ = 120 nm). This behavior suggests that Fe NWs exhibit different magnetization reversal regimes as a function of the Cu segments’ thickness.

In order to achieve a deeper understanding of the evolution of the easy magnetization axis and the Fe NWs magnetization reversal processes as a function of the Cu spacer thickness, FORC measurements were performed with the external magnetic field applied parallel to the NW long axis (Figure 3d–f). FORC diagrams clearly showed a striking behavior modification when increasing Cu thickness. 

In a first approximation, one could suggest that, for 15 nm Cu thickness, the NW internal dipolar coupling (between nanoelements inside a NW) was still sufficiently large to sustain a magnetization reversal process for the array similar to the one obtained in homogenous Fe NWs with comparable dimensions [24]. This hypothesis arises from the FORC distribution elongation along the *H*_u_ axis, typical of a demagnetizing mean interaction field (*H*_int_ = k*M/*M*_sat_, with the interaction field constant *k* < 0) and associated to the one-by-one NW reversal governed by this interaction field. 

A closer analysis of Figure 3d reveals that the FORC distribution exhibits two features: a slight counter-clockwise tilt and an additional elongation on the distribution right side, that are characteristic of a non-negligible coercivity distribution when submitted to a demagnetizing interaction field. In this case, the tilt arose from the fact that the magnetization reversal at negative *H_u_* values only involved the nanowires with the lowest coercivity. Following the procedure described in references [47,48], quantitative parameters could be estimated from the FORC diagram, based on its distribution extremities. In the present case, the experimental result was comparable to that of a system consisting of a normal distribution of coercivity ((750 ± 45) Oe) with a mean interaction field constant *k* = −800 Oe.

By increasing Cu spacing to 60 and 120 nm, the FORC distribution drastically changed into a single feature, elongated along the *H*_c_ axis and narrower as Cu thickness was increased (Figure 3e,f). The common explanation for this type of distribution is through a collection of non-interacting magnetic entities, for which the coercivity distribution can be extracted by the cross-section along the *H*_c_ axis [49]. In the specific context of multilayer magnetic NW arrays, it could indicate that Cu thickness between the Fe disks was sufficiently large to magnetically decouple them (along the NW axis), thus destroying the one-by-one NW reversal mode.

**Figure 4:**
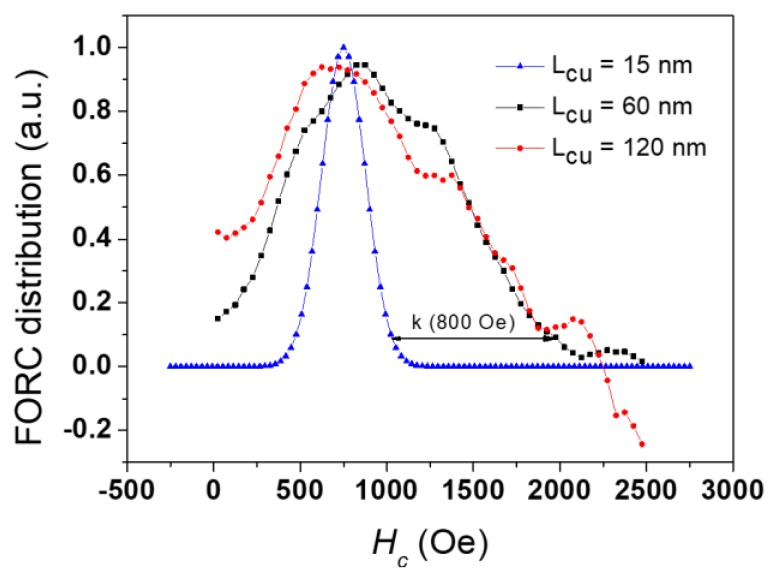
Smoothed and normalized cross sections along the *H*_c_ axis for Cu thicknesses of 60 (in black) and 120 nm (in red), along with the coercivity distribution estimated from the FORC distribution for 15 nm Cu thickness (in blue).

Since the dimensions and quantity of Fe discs remained identical in Figure 3e,f cases, it is interesting to compare their related *H*_c_ axis cross sections (See Figure 4). According to Preisach model, this cross section, taken as the FORC distribution values along the *H*_c_ axis, i.e., at *H*_u_ = 0 Oe, would directly yield the nanodisc coercivity distribution if the nanodiscs are completely decoupled one from each other (non-interacting). Because of the small amount of magnetic material, the comparison was complicated by the large noise-to-signal ratio present in the experimental results. However, it is possible to conclude that the two distributions are globally similar, i.e., that the modification in Cu thickness did not influence the Fe disc coercivity, as expected. On the other hand, when including the estimated coercivity distribution for the sample with 15 nm-thick Cu discs on the same graph, we observed an important reduction of the distribution width, while its maximum remained around the same value as for the samples with thicker Cu spacers. Whereas this value (≈750 Oe) is reasonable for Fe nanowires/nanodiscs of 45 nm in diameter, the *H*_c_ cross sections spread until coercivity values considerably higher than expected for these Fe nanodiscs.

Therefore, we propose an alternative hypothesis to explain the large elongation along the *H*_c_ axis when the multi-segmented NWs are decoupled. Instead of resulting solely from the individual nanodisc coercivity, this elongation may arise from the neighbor-to-neighbor nanodisc dipolar interaction. This interaction field is highly inhomogeneous inside the array, thus not yielding a mean interaction field behavior. Instead, the signature of the FORC distribution appears on the *H*_c_ axis at higher values than the nanodisc coercivity. This phenomenon is based on the same process that yields the common artifact of the “tail” towards high *H*_c_ values, visible on experimental NW FORC distributions and explained by Stancu et al. [50]. In this specific case, the NWs located near the array border were submitted to a reduced interaction field compared to the NWs near the array center, due to the lack of neighbors. This interaction field spatial inhomogeneity and, thus, the different values for the same applied field, resulted in an apparent higher reversal field for these NWs near the array border. The highest apparent reversal field value is the magnetic entity intrinsic coercivity added to the maximum interaction field, when the array is almost saturated. In the present case of multilayer NW arrays decoupled along the NW axis but creating a dipolar field on the neighbored discs, the intrinsic coercivity distribution of the Fe nanodiscs could be assumed to remain similar among the samples, despite the varying Cu nanodisc thickness. Therefore, the large elongation of their FORC distribution along the *H*_c_ axis arose from the same artifact explanation as for the homogeneous NW array.

### 3.4. Micromagnetic Simulations

In order to identify the magnetization reversal modes in Fe/Cu NWs and obtain a deeper insight into the effects of the magnetostatic interactions between segments and NWs, three-dimensional micromagnetic simulations using MuMax^3^ software (Version 3.9.1) [51] were performed. According to our experimental results, we simulated multi-segmented individual NWs with a Fe segment 40 nm in diameter and 35 nm in length and with non-magnetic Cu spacer lengths ranging from 15 to 120 nm. As the electrodeposited Fe could be contaminated by small amounts of Cu, the magnetization of the Fe layers was set to *M*_sat_ = 1600 emu/cm^3^, which was about 6% lower than Fe typical saturation magnetization value (1700 emu/cm^3^) [52]. This assumption was experimentally verified in multi-segmented Co/Cu NWs prepared by electrodeposition into AAO templates, where a saturation magnetization value of 1200 emu/cm^3^, instead of 1400 emu/cm^3^, was determined for Co and was justified by the presence of Cu impurities in Co [53]. We used an exchange coupling constant *A* = 43 × 10^−8^ erg/cm [54,55] and a Fe magnetocrystalline anisotropy value *K* = 4.8 × 10^5^ erg/cm^3^, based on the X-ray diffraction measurements. Cell size was chosen to be (2.5 × 2.5 × 2.5) nm^3^, which was two times smaller than Fe exchange length $l_{ex}=\sqrt{2A/\mu_{0}M_{sat}^{2}}\approx 5\;\mathrm{nm}$. Finally, the damping parameter was taken as 0.5 to ensure rapid convergence.

**Figure 5:**
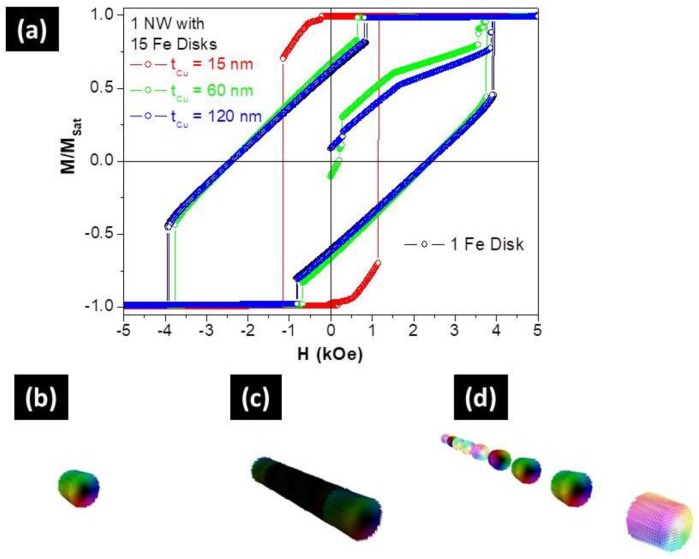
(**a**) Simulated hysteresis loops and virgin curves of one Fe disc (in black) and isolated multi-segmented Fe/Cu NWs with 15 discs of *L*_Fe_ = 35 nm separated by *L*_Cu_ = 15 (in red), 60 (in green), and 120 nm (in blue). 3D simulated magnetic configurations of (**b**) one Fe disc and isolated multi-segmented Fe/Cu NWs separated by *L*_Cu_ of (**c**) 15 nm and (**d**) 120 nm, at the demagnetization state.

First of all, the magnetostatic interactions and the magnetization reversal modes of an isolated Fe disc and multi-segmented Fe/Cu NWs were analyzed as a function of Cu thickness, with Fe layers ranging from 1 to 15. The simulated hysteresis loops of 1 and 15 Fe discs, separated by 120 nm of Cu spacer, were nearly identical (see Figure 5a). This fact confirmed our FORC analysis indicating that this segmented NW behaved like a set of 15 non-interacting nanoparticles. Moreover, in the demagnetized state, each Fe segment showed a kind of vortex configuration with around 60% of the magnetization pointed parallel to the NW long axis (Figure 5d), as in the case of an isolated Fe nanodisc (Figure 5b). 

On the other hand, the hysteresis loop of a NW with 15 Fe discs separated by 15 nm of Cu spacer, was drastically different than that of the isolated Fe disc, thus confirming that they were strongly coupled (Figure 5a). Moreover, and like in long cylindrical Fe NWs [56], the reversal mode corresponded to the nucleation and propagation of a vortex domain wall from the NW extremities, which was predicted from the FORC curves (Figure 3d). For the intermediate thickness (60 nm of Cu spacer), and although the hysteresis loop was similar to the non-interacting case, the Fe discs were still slightly coupled.

Finally, the effect of the magnetostatic interactions between NWs was explored by simulating NW arrays with a hexagonal closest packet ordering and 105 nm of periodicity. However, and because of time limitation, our simulations were restricted to 7 and 23 NW arrays and up to 3 Fe segments.

**Figure 6:**
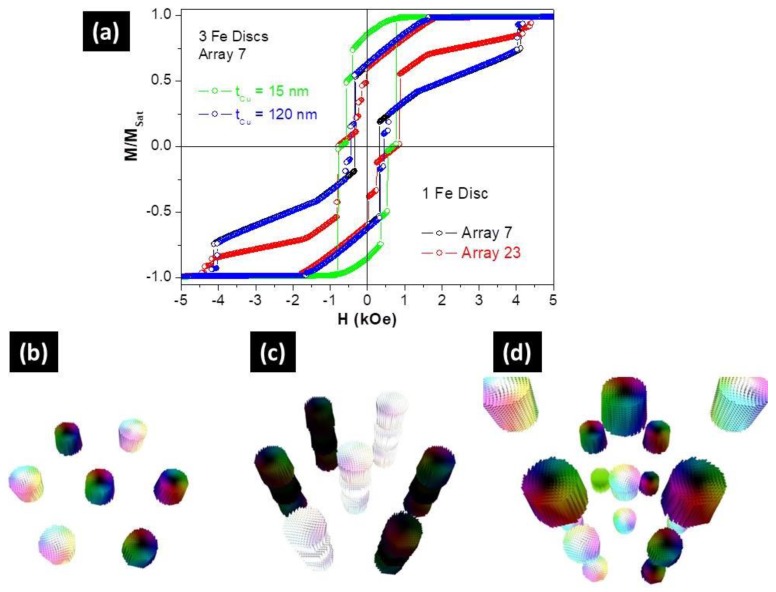
(**a**) Simulated hysteresis loops of one Fe disc array with 7 (in black) and 23 discs (in red), and seven multi-segmented nanowire arrays with three discs separated by *L*_Cu_ = 15 nm (in green) and 120 nm (in blue). 3D simulated magnetic configuration of one Fe disc array (**b**) and multi-segmented Fe nanowire arrays separated by *L*_Cu_ = 15 nm (**c**) and 120 nm (**d**) at the demagnetization state.

For the NWs array with one segment of Fe, the hysteresis loop showed a drastic dependence on the wire quantity (Figure 6a). Although the normalized remanence was not affected by the wire quantity, the coercivity was reduced from about 2.4 kOe for an isolated Fe segment to lower than 0.8 kOe for an array with 23 nanoelements. A similar behavior was observed for the NWs array with three Fe segments separated by 120 nm of Cu. Therefore, our simulations confirmed that the magnetostatic interaction between NWs governed the magnetic behavior of the Fe/Cu multi-segmented NWs formed by a set of non-interacting (along the axis) magnetic discs.

On the other hand, the hysteretic loop of a NW array with three Fe segments and *L*_Cu_ = 15 nm (Figure 6a), was similar to that of the isolated multi-segmented NW (Figure 5a) with small reductions of both coercivity and normalized remanence. In this case, we could conclude that the magnetic response was mainly controlled by the dipolar interactions between the Fe segments inside each NW.

Although simulations of the NW arrays allowed us to achieve good correlations with the experimental data, we should note that we studied a reduced number of NWs (7 or 23) and that the number of layers was also limited up to three elements. Therefore, the comparison with experiments, such as the coercive fields and remanence values, could be only quantitative. Simulations using arrays with a higher number of NWs and layers will be required for a more proper and qualitative analysis.

## 4. Conclusions

The present work shows that the pulsed electrodeposition method using a single bath successfully produced well-controlled multi-segmented NW arrays with magnetic (Fe) and non-magnetic (Cu) segments. The obtained length for the Fe segments was tuned to (30 ± 7) nm, while the Cu segments varied between (15 ± 5) nm and (120 ± 10) nm, with uniform layer thickness. A structural analysis revealed the formation of preferential Fe (110) crystalline directions in a body-centered cubic (bcc) structure. Magnetic measurements demonstrated that the Cu segments’ length played a role on the magnetization reversal regimes of the Fe NWs. FORC diagrams were obtained for all samples and showed that for Cu thicknesses of 15 nm, the system behaved like a NW array, while a decoupling of the Fe discs along the axis was observed for Cu spacer lengths greater than 60 nm. Regarding the extracted quantitative parameters, there was a normal distribution of coercivity ((750 ± 45) Oe) with a mean interaction field constant *k* = −800 Oe. 

According to our experimental results, we simulated multi-segmented individual NWs with a Fe segment 40 nm in diameter and 35 nm in length and with the non-magnetic Cu spacer length ranging from 15 to 120 nm. We concluded that the simulated hysteresis loops of one and 15 Fe discs, separated by 120 nm of Cu spacer, were nearly identical, which is in accordance with FORC analysis, where these segmented NWs behaved as a set of 15 non-interacting nanoparticles. On the other hand, the simulations also confirmed our FORC analysis where the Fe discs, separated by 15 nm of Cu spacer, were strongly coupled and behaved like a homogeneous Fe NW. Regarding the effect of the magnetostatic interactions between NWs, the hysteresis loop showed a drastic dependence on the number of wires for the NWs array with 120 nm of Cu spacer. The normalized remanence was not affected by the wire quantity, but the coercivity was significantly reduced when the number of NWs was increased. From these findings it is possible to conclude that the micromagnetic model can be used to describe the magnetic behavior of multi-segmented NWs.

## Figures and Tables

**Table 1:** Magnetic characteristics of the multi-segmented nanowires of (Fe_(Fe length)_/Cu_(Cu length)_) layers: coercive field (*H*_c_) and reduced remanence (*m*_r_) measured with the magnetic field applied parallel (||­) and perpendicular (­⊥) to the nanowires’ long axis.

Systems	$H_{c}^{\left|\right|}$(Oe)	$H_{c}^{⊥}$(Oe)	$m_{r}^{\left|\right|}$	$m_{r}^{⊥}$
(Fe_(30nm)_/Cu_(15nm)_)_15_	650 ± 50	190 ± 30	0.70 ± 0.03	0.04 ± 0.01
(Fe_(30nm)_/Cu_(60nm)_)_15_	480 ± 30	240 ± 40	0.45 ± 0.02	0.07 ± 0.02
(Fe_(30nm)_/Cu_(120nm)_)_15_	430 ± 30	280 ± 40	0.37 ± 0.02	0.20 ± 0.02
